# Supplementary material for: Smooth Interpolating Curves with Local Control and Monotone Alternating Curvature
Source: Comput Graph Forum. 2022 Oct 6;41(5):25–38. doi: 10.1111/cgf.14600 (PMC9827861; doi:10.1111/cgf.14600)
Supplement: Supplementary file 1 — Supplement Material [file CGF-41-25-s001.zip › Local-Smooth-Interpolating-MonoCurvature/extern/clothoids/docs/api-matlab/program_listing_file_CircleArc.m.html]

Program Listing for File CircleArc.m — Clothoids v2.0.9

### Navigation

- index
- toc
- Clothoids »
- Program Listing for File CircleArc.m

# Program Listing for File CircleArc.m¶

↰ Return to documentation for file (`CircleArc.m`)

```
classdef CircleArc < CurveBase
  %% MATLAB class wrapper for the underlying C++ class

  methods
    %> Create a new C++ class instance for the circle arc object
    %>
    %> **Usage:**
    %>
    %> \rst
    %> .. code-block:: matlab
    %>
    %>   ref = CircleArc() % create empty circle
    %>   ref = CircleArc( x0, y0, theta0, k0, L ) % circle passing from (x0,y0)
    %>                                            % at angle theta0 with curvature k0
    %>                                            % and length L
    %>
    %> \endrst
    %>
    %> **On input:**
    %>
    %> - `x0`, `y0`: coordinate of initial point
    %> - `theta0`:   orientation of the circle at initial point
    %> - `k0`:       curvature of the circle at initial point
    %> - `L`:        length of curve from initial to final point
    %>
    %> **On output:**
    %>
    %> - ref: reference handle to the object instance
    %>
    function self = CircleArc( varargin )
      self@CurveBase( 'CircleArcMexWrapper' );
      self.objectHandle = CircleArcMexWrapper( 'new', varargin{:} );
    end
    % - - - - - - - - - - - - - - - - - - - - - - - - - - - - - - - - - - -
    function str = is_type( ~ )
      str = 'CircleArc';
    end
    % - - - - - - - - - - - - - - - - - - - - - - - - - - - - - - - - - - -
    %>
    %> Build the circle from known parameters
    %>
    %> **Usage:**
    %>
    %> \rst
    %> .. code-block:: matlab
    %>
    %>    ref.build( x0, y0, theta0, k0, L )
    %>
    %> \endrst
    %>
    %> build a circle passing from (x0,y0) at angle theta0 with curvature and length
    %>
    %> **On input:**
    %>
    %> - `x0`, `y0`: coordinate of initial point
    %> - `theta0`:   orientation of the circle at initial point
    %> - `k0`:       curvature of the circle at initial point
    %> - `L`:        length of curve from initial to final point
    %>
    function build( self, x0, y0, theta0, k0, L )
      CircleArcMexWrapper( 'build', self.objectHandle, x0, y0, theta0, k0, L );
    end
    % - - - - - - - - - - - - - - - - - - - - - - - - - - - - - - - - - - -
    %>
    %> Build the circle from known parameters
    %>
    %> **Usage:**
    %>
    %> \rst
    %> .. code-block:: matlab
    %>
    %>    ref.build( x0, y0, theta0, x1, y1 ); % circle passing to [x0,y0] and [x1,y1]
    %>                                         % with angle theta0 at [x0,y0]
    %>    ref.build( p0, theta0, p1 ); % circle passing to p0 and p1 with angle theta0 at p0
    %>
    %> \endrst
    %>
    %> **On input:**
    %>
    %> - `x0`, `y0`: coordinate of initial point
    %> - `theta0`:   orientation of the circle at initial point
    %> - `k0`:       curvature of the circle at initial point
    %> - `L`:        length of curve from initial to final point
    %> - `p0`:       2D point
    %> - `p1`:       2D point
    %>
    function ok = build_G1( self, varargin )
      ok = CircleArcMexWrapper( 'build_G1', self.objectHandle, varargin{:} );
    end
    % - - - - - - - - - - - - - - - - - - - - - - - - - - - - - - - - - - -
    %>
    %> Build the circle arc given 3 points. The point can be alingned
    %> in this case a degenerate straight arc if build.
    %>
    %> **Usage:**
    %>
    %> \rst
    %> .. code-block:: matlab
    %>
    %>    ref.build_3P( x0, y0, x1, y1, x2, y2 )
    %>    ref.build_3P( p0, p1, p2 )
    %> \endrst
    %>
    function ok = build_3P( self, varargin )
      ok = CircleArcMexWrapper( 'build_3P', self.objectHandle, varargin{:} );
    end
    % - - - - - - - - - - - - - - - - - - - - - - - - - - - - - - - - - - -
    %>
    %> Scale circle by `sc` factor
    %>
    %> **Usage:**
    %>
    %> \rst
    %> .. code-block:: matlab
    %>
    %>    ref.scale( sc );
    %> \endrst
    %>
    function scale( self, sc )
      CircleArcMexWrapper( 'scale', self.objectHandle, sc );
    end
    % - - - - - - - - - - - - - - - - - - - - - - - - - - - - - - - - - - -
    %> Change the origin of the circle curve to `s0` and set arc lenght to `L`
    %>
    %> **Usage:**
    %>
    %> \rst
    %> .. code-block:: matlab
    %>
    %>    ref.changeCurvilinearOrigin( s0, L );
    %> \endrst
    %>
    function changeCurvilinearOrigin( self, s0, L )
      CircleArcMexWrapper( 'changeCurvilinearOrigin', self.objectHandle, s0, L );
    end
    % - - - - - - - - - - - - - - - - - - - - - - - - - - - - - - - - - - -
    %> return a nurbs representation of the circle arc
    function nurbs = to_nurbs( self )
      nurbs = CircleArcMexWrapper( 'to_nurbs', self.objectHandle );
    end
    % - - - - - - - - - - - - - - - - - - - - - - - - - - - - - - - - - - -
    %> Plot the arc
    %>
    %> **Usage:**
    %>
    %> \rst
    %> .. code-block:: matlab
    %>
    %>   ref.plot( npts );
    %>
    %>   fmt = {'Color','blue','Linewidth',2};
    %>   ref.plot( npts, fmt );
    %>
    %> \endrst
    %>
    %> - `npts`: number of sampling points for plotting
    %> - `fmt` : format of the arc
    %>
    function plot( self, npts, fmt )
      if nargin<2
        npts = 128;
      end
      if nargin<3
        fmt = {'Color','blue','Linewidth',2};
      end
      L = self.length();
      S = 0:L/npts:L;
      [X,Y] = self.eval(S);
      plot(X,Y,fmt{:});
    end
    % - - - - - - - - - - - - - - - - - - - - - - - - - - - - - - - - - - -
    %> Plot the polygon of the NURBS for the arc
    %>
    %> **Usage:**
    %>
    %> \rst
    %> .. code-block:: matlab
    %>
    %>   ref.plotPolygon();
    %>   ref.plotPolygon( 'Color','blue','Linewidth',2 );
    %>
    %> \endrst
    %>
    %> - `fmt` : format of the arc
    %>
    function plotPolygon( self, varargin )
      arc = self.to_nurbs();
      xx  = arc.coefs(1,:)./arc.coefs(3,:);
      yy  = arc.coefs(2,:)./arc.coefs(3,:);
      if nargin > 1
        plot(xx,yy,varargin{:});
      else
        plot(xx,yy,':ok');
      end
    end
  end
end
```

### Quick search

### Table of Contents

- Matlab Interface Manual
- C++ API
- MATLAB API

«
hide menu

menu
sidebar
»

### Navigation

- index
- toc
- Clothoids »
- Program Listing for File CircleArc.m

© Copyright 2021, Enrico Bertolazzi and Marco Frego.
Created using Sphinx 4.2.0.
